# Integrated analysis of long non-coding RNAs (lncRNAs) and mRNA expression profiles identifies lncRNA PRKG1-AS1 playing important roles in skeletal muscle aging

**DOI:** 10.18632/aging.203067

**Published:** 2021-05-29

**Authors:** Yan Zheng, Ting Liu, Qun Li, Jie Li

**Affiliations:** 1 Department of Geriatrics, The First Hospital of Jilin University, Changchun 130021, Jilin, P.R. China; 2 Department of Thyroid Surgery, The First Hospital of Jilin University, Changchun 130021, Jilin, P.R. China

**Keywords:** long non-coding RNAs, skeletal muscle aging, differentially expressed genes, PRKG1-AS1

## Abstract

This study aimed to identify long non-coding RNAs (lncRNAs) involving in the skeletal muscle aging process. Skeletal muscle samples from old and young subjects were collected for lncRNA-sequencing. Differentially expressed genes (DEGs) and DElncRNAs between young and old groups were identified and a co-expression network was built. Further, a dexamethasone-induced muscle atrophy cell model was established to characterize the function of a critical lncRNA. A total of 424 DEGs, including 271 upregulated genes and 153 downregulated genes as well as 152 DElncRNAs including 76 up-regulated and 76 down-regulated lncRNAs were obtained. Functional analysis demonstrated that the DEGs were significantly related to immune response. Coexpression network demonstrated lncRNA AC004797.1, PRKG1-AS1 and GRPC5D-AS1 were crucial lncRNAs. Their expressions were further validated by qRT-PCR in human skeletal muscle and the muscle atrophy cell model. Further *in vitro* analysis suggested that knock-down of PRKG1-AS1 could significantly increase cell viability and decrease cell apoptosis. qRT-PCR and western blot analyses demonstrated that knock-down of PRKG1-AS1 could increase the expression of MyoD, MyoG and Mef2c. This study demonstrated that lncRNAs of GPRC5D-AS1, AC004797.1 and PRKG1-AS1 might involve the aging-associated disease processes.

## INTRODUCTION

Population aging is occurring throughout the world. In the 21st century, Europe will continue to have the world's oldest population, and by 2030, nearly a quarter of Europeans will reach 65 years old or over [1]. Aging as a complex phenomenon is the progressive and inevitable process of bodily deterioration in growing elderly population [2]. Skeletal muscle, which is one of the major organs responsible for body movements and metabolism is one of the earliest tissues to be affected by aging [3]. Skeletal muscle abnormalities are responsible for tissue homeostasis, functional impairment, loss of mass, sarcopenia and disability in the elderly [4, 5]. About 0.5% - 1% of muscle mass loss is lost every year in people over 30 years of age, and the rate of decline is rapidly increasing after 65 years old [6]. It is estimated that the incidence of sarcopenia in 60 year or above age group is 5% - 13%, and that in 80 year or above age group increased to 50% [7]. A better understanding of molecular modulation mechanism in skeletal muscle aging is imperative to ameliorate the problem in a rapidly aging population.

Along with the aging process, skeletal muscle mass and strength gradually decline [8], which might further result in muscle atrophy. Increased protein degradation and decreased protein synthesis along with loss of innervation of aging myofibers occurred in aging humans [9]. Aging process is driven by changes in expression of a large number of genes. A variety of noncoding RNAs (ncRNAs), both short ncRNAs (mainly microRNAs, miRNAs) and long ncRNAs (lncRNAs) are reported to regulate gene expression at the post-transcriptional level. Our previous report indicated that two candidate miRNAs (miR-19a and miR-34a) might play regulatory roles in the aging process of skeletal muscles [10]. Currently, the regulatory mechanisms of lncRNAs with known or unknown function were reported in aging mammals [11, 12]. A recent study reviewed the lncRNAs associating with age-related muscle pathology and suggested that lncRNAs affect aging-impaired proliferative and immune responses as well as modulate age-related neurodegeneration [13]. They suggested that lncRNAs might be promising therapeutic targets for diseases related with aging, such as hypertension, diabetes, Alzheimer's disease, Parkinson's disease and cancer [13]. The regulatory mechanisms of some lncRNAs have also been reported. Neppl et al. reported that lncRNA Chronos is an aging-induced lncRNA, which could induce myofiber atrophy when overexpressed [14]. LncRNA *H19* may participate in skeletal muscle regeneration via interacting with let-7 [15, 16]. Muscle-specific *linc-MD1* could be interacted with HuR to function in muscle regeneration [17, 18]. LncRNA *Dum* activates myogenesis via silencing a repressor of myogenesis (DPPA2, developmental pluripotency-associated 2) [19], and lncRNA MALAT1, which is associated with proliferation of myoblasts and endothelial cells, may be a regulator of myogenesis during muscle aging [20, 21]. LncDLEU2 might inhibit muscle differentiation and regeneration by acting as a miR-18a sponge to regulate SEPP1 [22]. The comprehensive transcriptional landscape of lncRNAs associated with skeletal muscle aging was fewly investigated. Chen et al. performed high throughput RNA sequencing on skeletal muscles in different age conditions and identified 5 differentially expressed lncRNAs (DElncRNAs) related with skeletal muscle aging [23]. However, the expression levels of them were not further validated, nor the molecular mechanism was explored.

To better understand the biological roles of lncRNAs in conditions of skeletal muscle aging, we performed lncRNA sequencing on skeletal muscle samples from old and young subjects. The lncRNA expression data were integrated with differentially expressed mRNA (DEGs) data to identify skeletal muscle aging-related lncRNAs and genes. Further, a dexamethasone-induced muscle atrophy cell model was established to characterize the function of a critical lncRNA. In summary, the predicted lncRNAs and genes involving in the potential mechanisms of muscle aging can be utilized in further studies of preventing muscle aging.

## RESULTS

### Overview of RNA-sequencing and identification of mRNA and lncRNAs in old group

The RNA-Sequencing data from 6 subjects were analyzed and a total of 583,406,044 raw reads were obtained. After quality control, 582,678,196 clean reads were left. The base average error rate of clean reads was 0.024%, and the average Q20 and Q30 values were 98.48 and 95.24%, respectively. The average GC content was 47.9% (Table 1).

**Table 1:** Summary of sequencing quality.

**Samples**	**Raw reads**	**Clean reads**	**Clean bases (bp)**	**Error%**	**Q20%**	**Q30%**	**GC%**
Old 1	88089696	87982698	12619031100	0.0242	98.33	95	49.11
Old 2	90157914	90082856	12818911171	0.0237	98.58	95.46	48.34
Old 3	116152404	115990710	16621722517	0.0236	98.62	95.54	47.85
Young 1	89679340	89476230	12716644800	0.024	98.4	95.16	46.73
Young 2	102798714	102703152	14984574299	0.0237	98.6	95.48	47.39
Young 3	96527976	96442550	13850012035	0.0243	98.35	94.78	47.98
Summary	583406044	582678196	83610895922				

**Figure 1:**
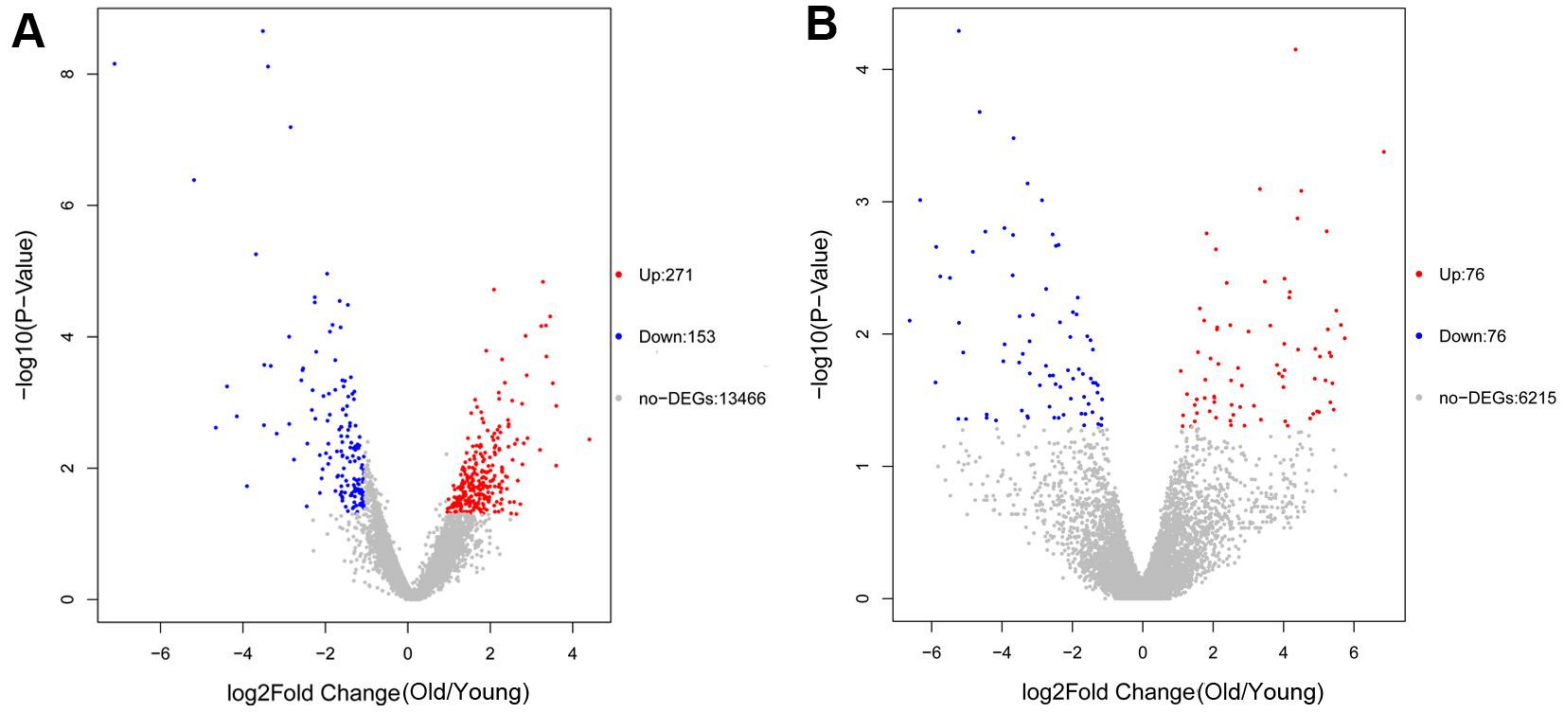
Volcano plot of differentially expressed genes (DEGs) (**A**) and differentially expressed lncRNAs (DElncRNAs) (**B**). The red dots represent upregulated genes or lncRNAs and blue dots represent downregulated genes or lncRNAs.

A total of 424 DEGs were identified, including 271 up-regulated and 153 down-regulated genes (Figure 1A, Supplementary Table 1). In addition, 152 DElncRNAs including 76 up-regulated and 76 down-regulated genes were obtained (Figure 1B and Supplementary Table 2).

### Functional enrichment analyses of DEGs

**Figure 2:**
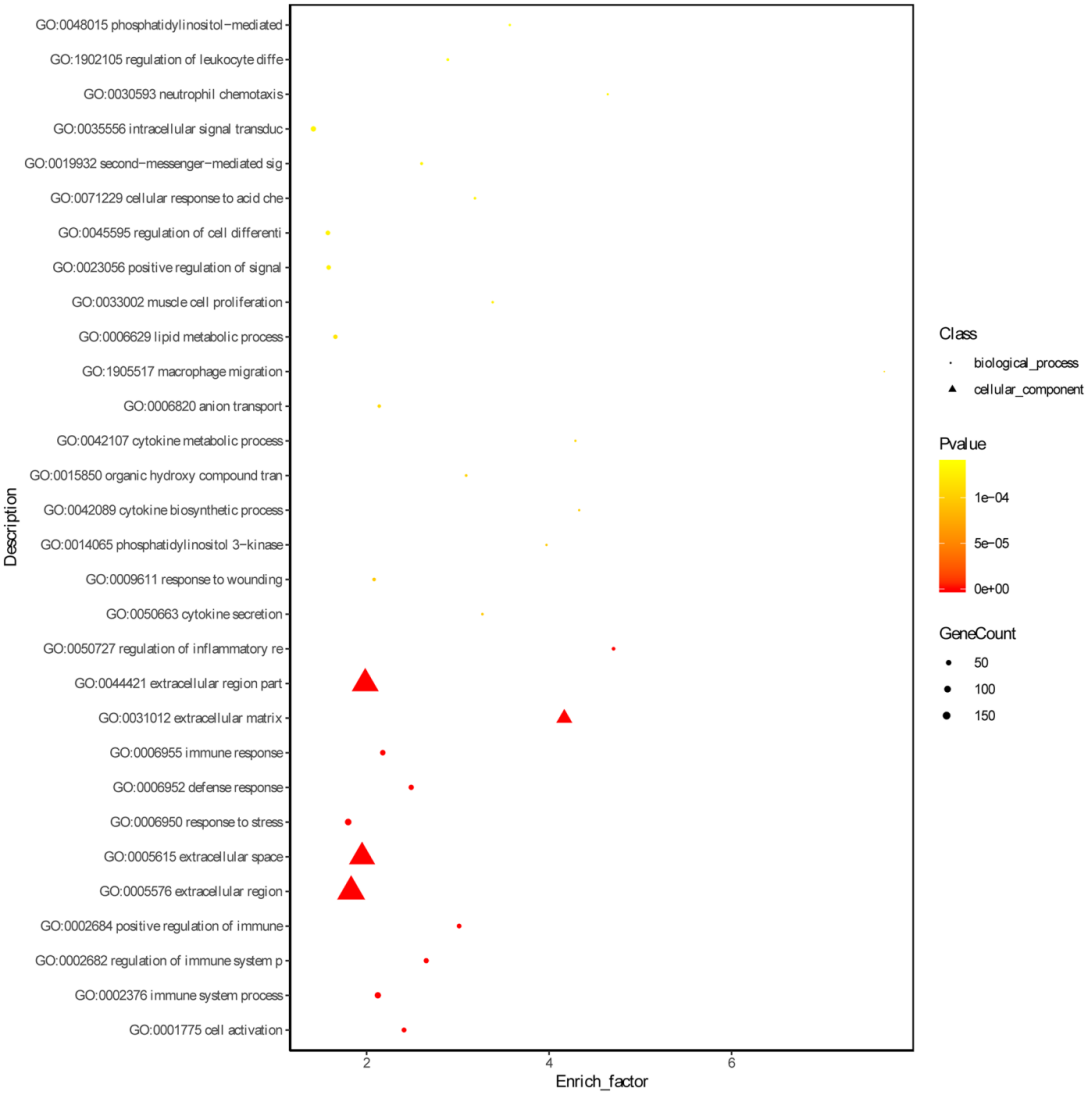
**Gene ontology enrichment analyses of DEGs.** ‘Round’ represents biological process term and ‘tri-angle’ represents cell component term.

**Figure 3:**
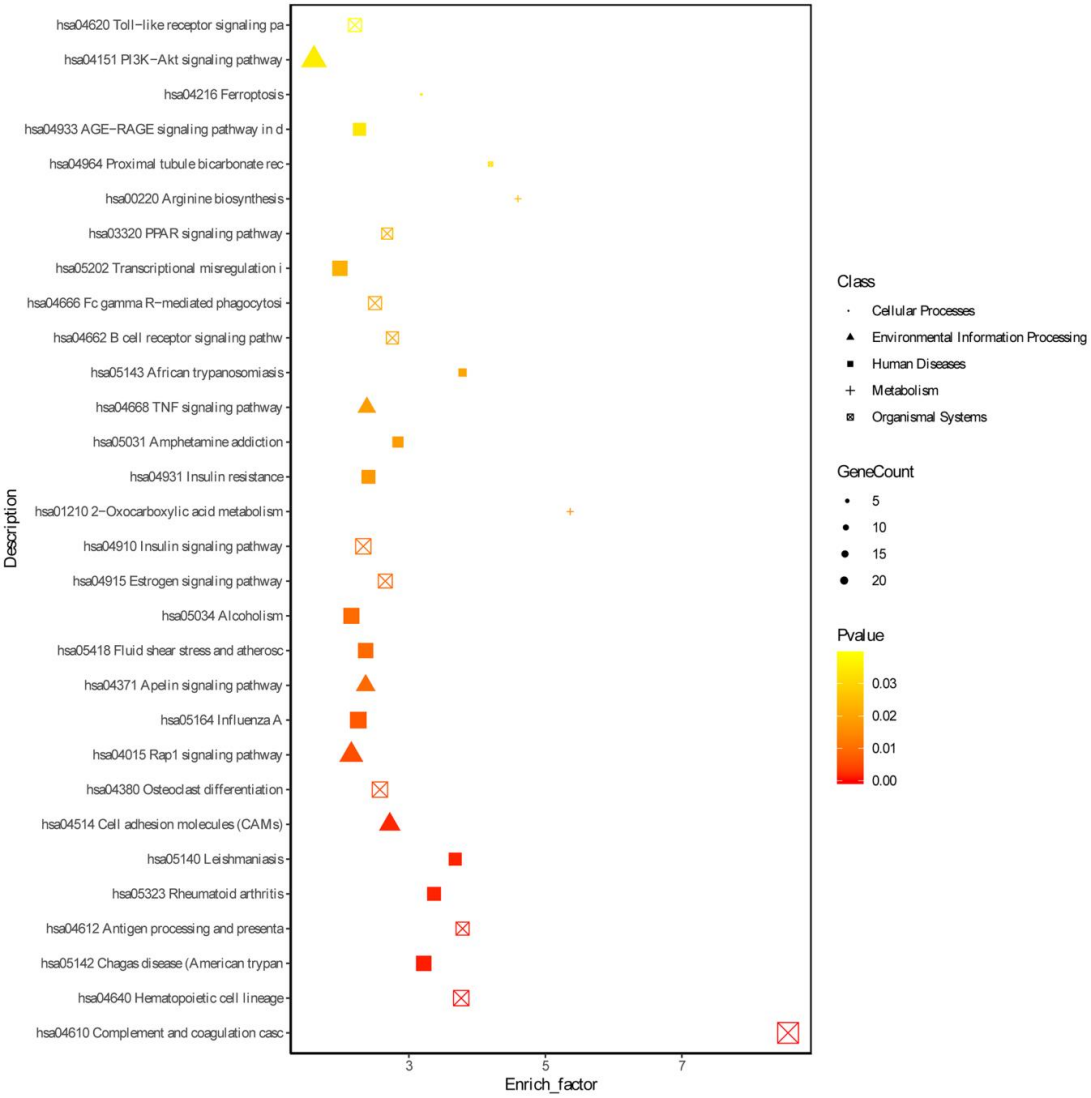
**KEGG enrichment analyses of DEGs.** ‘Round’ represents cellular processes term, ‘tri-angle’ represents environmental information processing, ‘square’ represents human diseases, ‘plus sign’ represents metabolism term and ‘cross within square’ represents organismal systems term.

Based on the threshold of FDR < 0.05, we obtained 772 GO terms for the DEGs. The top significant GO terms were related with immune response, such as “immune system process” (gene count: 133; FDR = 0) and “regulation of immune system process” (gene count: 84; FDR = 0) and “immune response” (gene count: 94; FDR = 0) (Figure 2, Supplementary Table 3). Simultaneously, we obtained 15 significant KEGG pathways for the DEGs, including “Complement and coagulation cascades” (gene count = 21, FDR = 0), “Phagosome” (gene count = 15; FDR =0.0016), and hematopoietic cell lineage (gene count = 11; FDR = 0.0037) (Figure 3 and Supplementary Table 4).

### Co-expression network of DElncRNAs and DEGs

**Figure 4:**
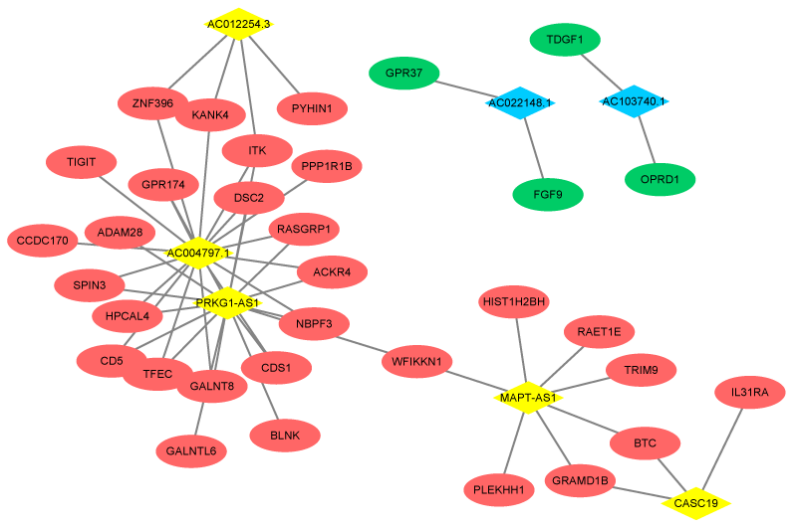
**Co-expression network of DEGs and DElncRNAs.** The red ellipse represent upregulated gene and the green ellipse represent downregulated gene. The yellow rhombus represents upregulated lncRNA and blue rhombus represents downregulated lncRNA. The lines between the genes and lncRNAs indicate that there is a co-expression relationship between the two.

Based on the Pearson correlation coefficient > 0.9, a co-expression network containing 7 DElncRNAs, 33 DEGs and 51 edges was built (Figure 4). Five upregulated lncRNAs, including AC004797.1 (degree = 17), PRKG1-AS1 (Protein kinase CGMP-dependent 1-antisense 1; degree = 16), MAPT-AS1 (microtubule associated protein tau –antisense 1; degree = 7), AC012254.3 (degree = 4) and CASC19 (degree = 3) and two downregulated lncRNAs, including AC022148.1 (degree = 2) and AC103740.1 (degree = 2) were included in this network. The DEGs of *ITK* (IL2 inducible T cell kinase) and *DSC2* (desmocollin 2) could be positively regulated by the lncRNAs AC004797.1 and PRKG1-AS1.

### Validation of key genes by qRT-PCR

**Figure 5:**
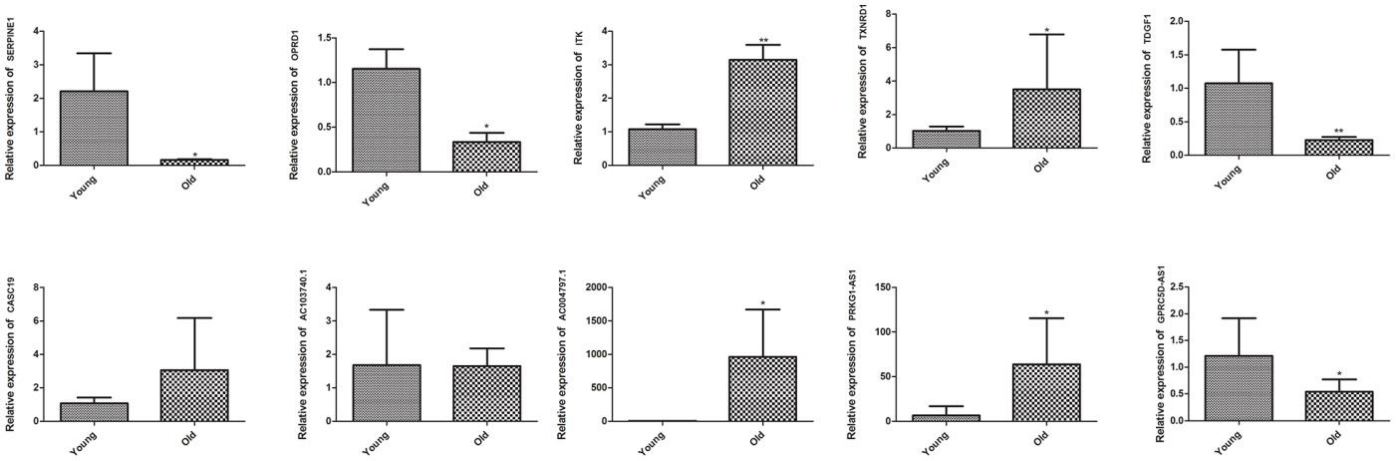
**Validation of key DEGs and DElncRNAs by quantitative real-time polymerase chain reaction (qRT-PCR) in skeletal muscle of young group and old group.** Difference between young group and old group was analyzed by students’ t test. * *P* < 0.05, ** *P* < 0.01.

To further validate the reliability of RNA sequencing, we performed qRT-PCR on five DEGs, including *SERPINE1* (Serpin family E member 1), *OPRD1* (Opioid receptor delta 1), *ITK*, *TXNRD1* (Thioredoxin reductase 1) and *TDGF1* (Teratocarcinoma-derived growth factor 1), as well as five DElncRNAs, including CASC19, AC103740.1, AC004797.1, PRKG1-AS1 and GPRC5D (G protein-coupled receptor class c group 5 member D)-AS1. The RNA sequencing data showed that *ITK*, *TXNRD1*, CASC19, AC004797.1, and PRKG1-AS1 were upregulated, while *SERPINE1*, *OPRD1*, *TDGF1*, AC103740.1 and GPRC5D-AS1 were downregulated in the muscle of old group. As shown in Figure 5, the qRT-PCR results of *ITK*, *TXNRD1*, AC004797.1, PRKG1-AS1, *SERPINE1*, *OPRD1*, *TDGF1*, and GPRC5D-AS1 were in line with the RNA sequencing data, while no significant difference on CASC19 and AC103740.1 were detected between muscle of young and old group by qRT-PCR.

### Establishment of a dexamethasone-induced muscle atrophy cell model

**Figure 6:**
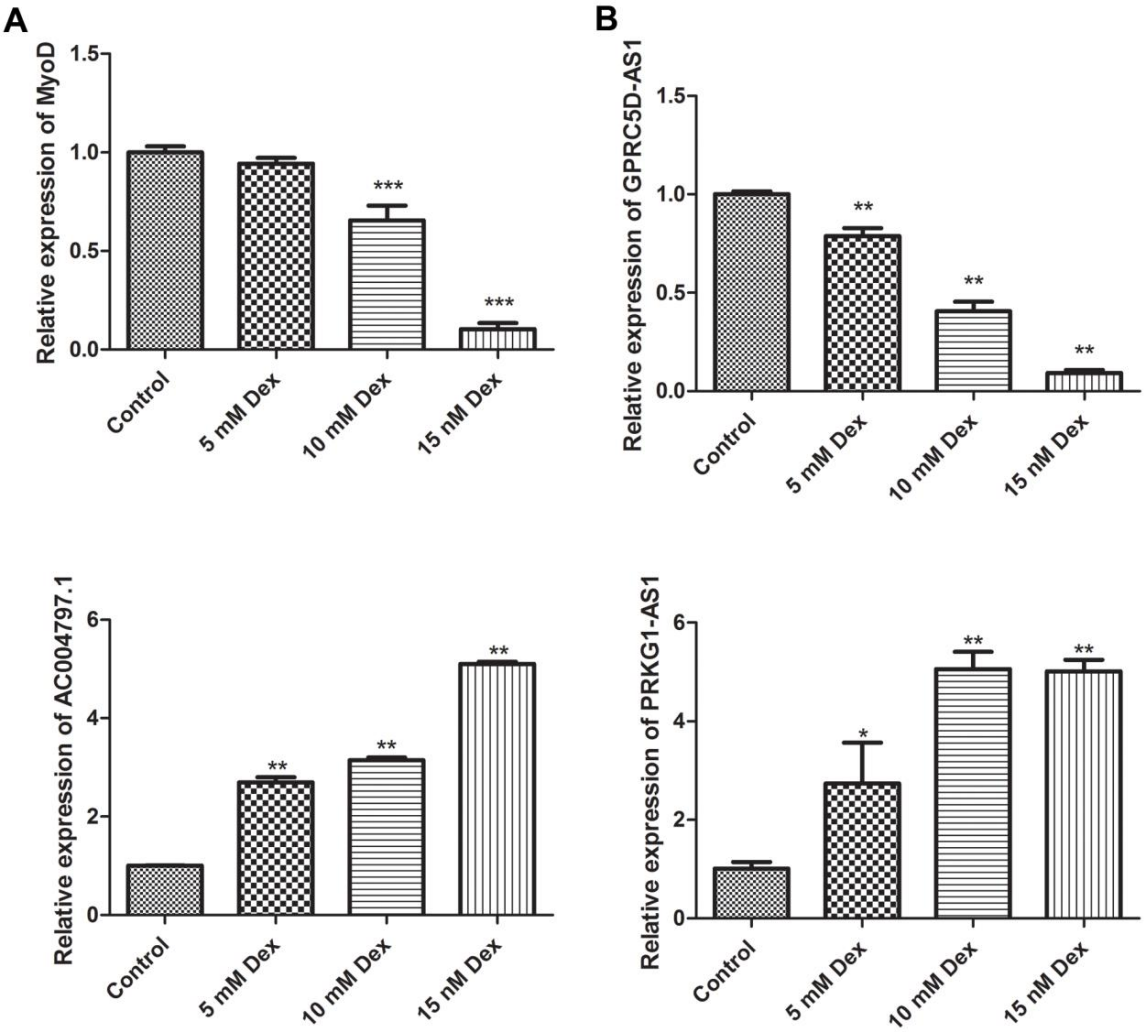
Validation of MyoD (**A**) and key DElncRNAs (**B**) by qRT-PCR in a dexamethasone-induced muscle atrophy cell model. Different concentrations of dexamethasone (Dex, 5 mM, 10 mM and 15 mM) were added in human skeletal muscle myoblasts and incubated for 48 h. Difference among groups was analyzed by ANOVA with Dunnett’s multiple comparison test. * *P* < 0.05, ** *P* < 0.01, ***P < 0.001, compared with control group.

To further investigate the roles of critical DElncRNAs, we established a muscle atrophy cell model by dexamethasone. The expression of myoblast determination protein 1 (MyoD), a skeletal muscle-specific bHLH transcription factor, was gradually decreased in a dose-dependent manner. The expression of MyoD at 10 nM and 15 nM dexamethasone treatment groups was significantly different from that of control group (*P* < 0.001), indicating the muscle atrophy cell model was successfully established (Figure 6A). The expression levels of three DElncRNAs, including PRKG1-AS1, AC004797.1 and GPRC5D-AS1 were determined under dexamethasone treatment. As expect, the expression levels of PRKG1-AS1 and AC004797.1 was increased in a dose-dependent manner, while GPRC5D-AS1 was decreased in a dose-dependent manner (*P* < 0.05, Figure 6B).

### Knockdown of PRKG1-AS1 increased cell viability and decreased cell apoptosis

**Figure 7:**
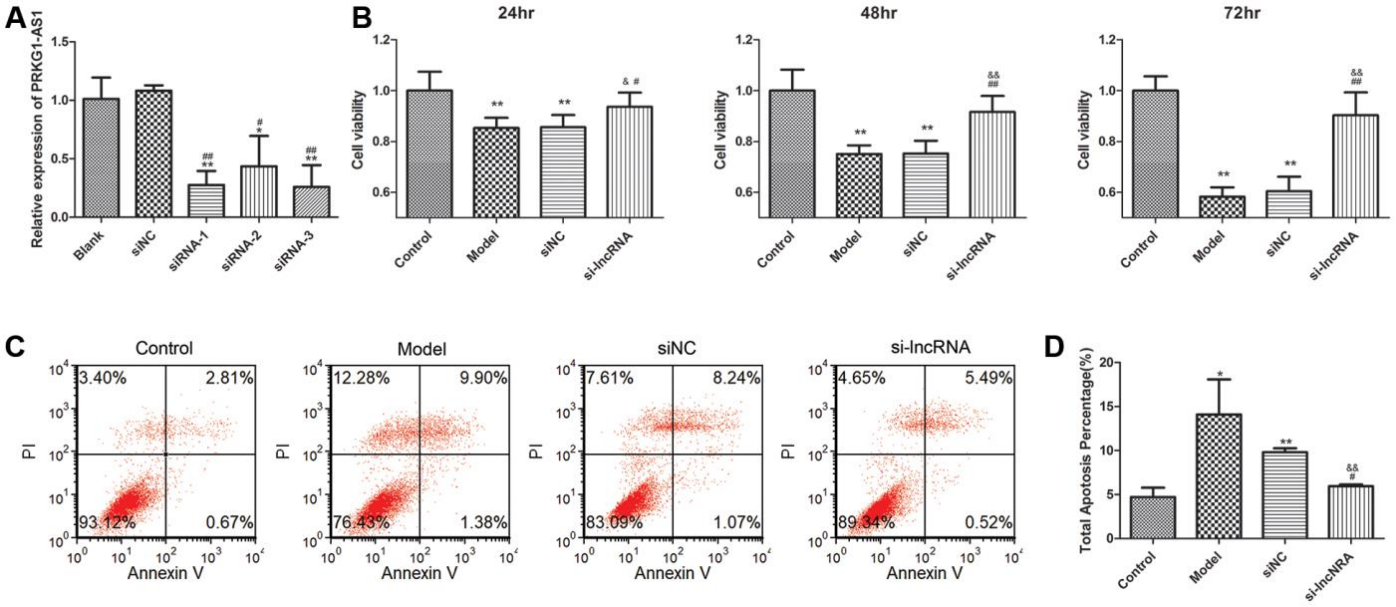
**The effect of knock-down of PRKG1-AS1 on cell viability and cell apoptosis.** (**A**) PRKG1-AS1 was knocked down by small interference RNA (siRNA) and the efficiency was detected by qRT-PCR. Difference among groups was analyzed by ANOVA with Bonferroni’s multiple comparison test. * *P* < 0.05, ** *P* < 0.01 compared with blank group; # *P* < 0.05, ## *P* < 0.01 compared with siNC (negative control) group. (**B**) Cell viability was tested by CCK-8 assay. Dexamethasone (15 mM) was added in human skeletal muscle myoblasts to establish atrophy cell model. Si-PRKG1-AS1 or siNC was transfected into human skeletal muscle myoblasts and incubated for 24 h, 48 h and 72 h. Cell viability was tested by CCK-8 assay. ** *P* < 0.01 compared with control group; # *P* < 0.05, ## *P* < 0.01 compared with model group; & *P* < 0.05, && *P* < 0.01 compared with siNC group. (**C**) Cell apoptosis was tested by flow cytometry. Dexamethasone (15 mM) was added in human skeletal muscle myoblasts to establish atrophy cell model. Si-PRKG1-AS1 or siNC was transfected into human skeletal muscle myoblasts and incubated for 48 h. (**D**) Quantitative analysis of cell apoptosis. * *P* < 0.05, ** *P* < 0.01 compared with control group; # *P* < 0.05 compared with model group; && *P* < 0.01 compared with siNC group.

We selected PRKG1-AS1 for further research. The expression of PRKG1-AS1 was knocked down by siRNA. qRT-PCR suggested that the expression of PRKG1-AS1 in siRNA1, siRNA2 and siRNA3 was all decreased and the decrease of PRKG1-AS1 in siRNA3 group was the most significant (*P* < 0.01, Figure 7A). Therefore, we selected siRNA3 for further experiment. CCK-8 assay showed that knock-down of PRKG1-AS1 could significantly increase cell viability in a time-dependent manner (*P* < 0.05, Figure 7B). Besides, flow cytometry demonstrated that cell apoptosis was significantly decreased by knocking-down of PRKG1-AS1 compared with the model group (*P* < 0.01, Figure 7C, 7D).

### Knockdown of PRKG1-AS1 affected mRNA and protein expression of muscle regulatory factors

**Figure 8:**
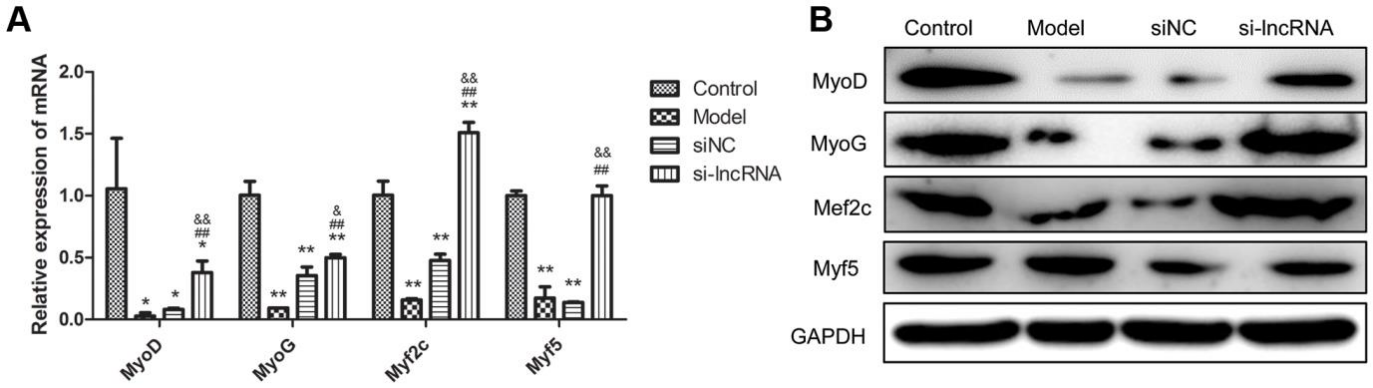
**The effect of knock-down of PRKG1-AS1 on muscle regulatory factors.** (**A**) qRT-PCR analyzes gene expression of MyoD, MyoG, Myf2c and Myf5. Dexamethasone (15 mM) was added in human skeletal muscle myoblasts to establish atrophy cell model. Si-PRKG1-AS1 or siNC was transfected into human skeletal muscle myoblasts and incubated for 48 h. * *P* < 0.05, ** *P* < 0.01 compared with control group; ## *P* < 0.01 compared with model group; & *P* < 0.05, && *P* < 0.01 compared with siNC group. (**B**) Protein expression of MyoD, MyoG, Myf2c and Myf5 detected by western blot. Dexamethasone (15 mM) was added in human skeletal muscle myoblasts to establish atrophy cell model. Si-PRKG1-AS1 or siNC was transfected into human skeletal muscle myoblasts and incubated for 48 h.

In order to validate the effect of PKG1-AS1 at molecular level, we detected the mRNA and protein expression of muscle regulatory factors, including MyoD, MyoG, Myf2c and Myf5. As shown in Figure 8, these four factors were significantly decreased in the dexamethasone-induced muscle atrophy cell model (*P* < 0.05). After transfection with si-PRKG1-AS1, their expression was significantly upregulated both at mRNA level (*P* < 0.05). Western blot analysis showed consistent results with qRT-PCR, except for myf5, which showed no significant difference among groups.

## DISCUSSION

Skeletal muscle is one of the earliest tissues being affected by aging. Therefore, investigating the molecular mechanism that governs aging-associated pathologies is imperative to ameliorate the problem in a rapidly aging population. This study performed lncRNA sequencing on skeletal muscle samples from old and young subjects and analyzed the candidate genes and lncRNAs that involve skeletal muscle aging by integrating analysis on lncRNAs and mRNA profiles. We identified 424 DEGs and 152 DElncRNAs that might be related with skeletal muscle aging. Functional analysis demonstrated the DEGs were significantly related to immune response. Further, qRT-PCR validated the lncRNA GPRC5D-AS1 and the genes *SERPINE1*, *OPRD1*, *TDGF1* were significantly decreased, while lncRNAs of AC004797.1 and PRKG1-AS1, as well as genes *TXNRD1* and *ITK* were increased during skeletal muscle aging process.

Functional analysis demonstrated the DEGs were significantly related to immune response, such as “immune system process”, “regulation of immune system process” and “Complement and coagulation cascades”. Chronic inflammation induced organ damage is one of the major risk factors for age-related chronic diseases, such as Alzheimer's disease, atherosclerosis, diabetes, sarcopenia and cancer, and this is the result of a life-long active immune system [24]. Immune activation increases with age, including plasma C-reactive protein, interleukin-6, and tumor necrosis factor receptor, involves in regulation of skeletal muscle protein balance and decrease of protein synthetic rates, including the myosin heavy chain (MHC) protein [25]. Dysregulation of lncRNAs have been reported to participate in regulating innate immune responses in aging process [26]. However, only limited lncRNAs, such as linc-MAF-4, have been identified previously in post-transcriptionally regulating CD4+T- cell subsets [27] and no direct or indirect evidence point to their involvement in aging. Our results found two inflammatory and immune-related genes, including *ITK* and *OPRD1* were differentially expressed between young and old groups. *ITK* encodes interleukin 2-inducible T cell kinase, which involves in adaptive immune response and growth, signaling and function of myeloid-, mast-, B-cells and T-cell. *ITK* regulates IL-8, overproduction of which associated with oxidative stress via oxidative inactivation of the proteasome [28]. However, there is little report on the role of *ITK* in skeleton muscle aging currently. In the co-expression network, *ITK* was found being positively regulated by AC004797.1 and PRKG1-AS1. GO enrichment analysis suggested *ITK* participated in “Cell activation”, “immune system process”, “regulation of immune system process”, “positive regulation of immune system process”, etc. This result suggested that AC004797.1 and PRKG1-AS1 might participate in regulating immune response in skeletal muscle aging by targeting *ITK*. *OPRD1* associated with opioid dependence is also involved in innate immune system [29]. GO enrichment analysis in our study suggested *OPRD1* was enriched in terms of immune system process and response to stress. There was no direct report of the relationship between *OPRD1* and muscle aging. However, lncRNA NONMMUT000384 which is differentially expressed in mice of different ages could be functional annotated by *OPRD1* [30].

Dexamethasone is a glucocorticoid that could affect the synthesis and degradation of muscle proteins [31]. Excess dexamethasone causes muscle atrophy by inhibiting protein synthesis of myogenic transcription factors, such as MyoD and promoting protein degradation, and therefore induces muscle atrophy [32]. Dexamethasone-induced muscle atrophy is an excellent model to mimic muscle atrophy investigation [33]. Along with aging process, skeletal muscle mass and strength decrease. This decrease is referred to as skeletal muscle atrophy. Therefore, we established a dexamethasone-induced muscle atrophy cell model to validate the dysregulated lncRNAs in aging process. Skeletal muscle differentiation is mediated by a number of transcription factors, including members in myogenic regulatory family (MRF) and those in myocyte enhancer 2 family (MEF2) [34]. The MRF family including MyoD, MyoG, Myf5 and MRF4, function in regulating gene transcription in muscle cells, cell growth cycle and differentiation [35]. MyoD is a skeletal muscle-specific bHLH transcription factor that participated in muscle differentiation and repair [36]. The expression of MyoD was gradually decreased in a dose-dependent manner, indicating the muscle atrophy cell model was successfully established. *In vitro* experiments demonstrated that knock-down of PRKG1-AS1 could significantly increase cell viability and decrease cell apoptosis as well as increase the expression of muscle regulatory factors, including MyoD, MyoG and Mef2c. These results partly confirmed the role of PRKG1-AS1 in skeletal muscle aging.

*SERPINE1* which encodes a member of the serine proteinase inhibitor (serpin) superfamily were found involved in the KEGG pathways of “complement and coagulation cascades” and “p53 signaling pathway”, and GO terms of “defense response”, “inflammatory response” and “immune system process” in our study. SERPINE1 participates in fibrosis [37] and was identified as an antiangiogenic factor [38]. It is induced in response to elevated reactive oxygen species contributed by transforming growth factor-β1 stimulation [39]. Khan et al. indicated that *SERPINE1* encodes plasminogen activator inhibitor-1 with a null mutation could protect against biological aging and play a role in metabolism in humans [40]. In addition, *SERPINE1* was also identified by Shafiee et al. [41] and Ji et al. [42] in identifying of candidate genes in skeletal muscle with aging. No further studies were done in their studies; however, the expression of *SERPINE1* could be increased by knockdown of *SRGN*, which may interact with secreted factors and regulate storage or secretion in human skeletal muscle [43].

In addition, our results showed that *TXNRD1*, which is one of the nitric oxide synthase (NOS) family enzymes and the reactive oxygen species clearance enzymes, was enriched in GO terms of “response to stress”. *TXNRD1* which is essential for cellular function, cell proliferation and antioxidant defense is decreased during aging [44], and mammalian aging may be partly as a result of cellular redox state [45]. Cytosolic *TXNRD1* was found lowly expressed in old Nrf2-/- mice than those in young or old wild type controls. Besides, Nrf2 deficiency exacerbates age-related loss of skeletal muscle mass [46].

There are currently no direct reports on TDGF1 and muscle aging. TDGF1 involves in differentiation of mesoderm [47], and it was found lowly expressed response to oxidative stress induced by paraquat [48]. The TDGF1 protein is one of the markers characterizing pluripotent human embryonic stem cells [49] which is promising for treating aging-associated diseases. Our study showed that TDGF1 was enriched into GO terms of “defense response”, “immune system process” and “positive regulation of response to stimulus”, and it was being positively regulated by the DElncRNA AC103740.1.

In conclusion, we identified 424 DEGs and 152 DElncRNAs that might be related with skeletal muscle aging, including *SERPINE1*, *OPRD1*, *TDGF1*, *TXNRD1* and *ITK* as well as GPRC5D-AS1, AC004797.1 and PRKG1-AS1. We found the lncRNAs of AC004797.1 and PRKG1-AS1 might involve in skeletal muscle aging via, at least to some extent, the immune-inflammatory pathways. Knock-down of PRKG1-AS1 could significantly increase cell viability and decrease cell apoptosis. qRT-PCR and western blot analyses demonstrated that knock-down of PRKG1-AS1 could increase the expression of MyoD, MyoG and Mef2c. The lncRNAs and genes identified in this study might be promising candidates to limit aging-associated disease processes.

## MATERIALS AND METHODS

### Ethics statement

The study was performed according to protocols approved by the Ethics Committee of First Hospital of Jilin University (Changchun, China). Written informed consent for participating in this study has been received from all subjects.

### Sample collection

The old and young skeletal muscle from 6 subjects (age, 17–81 years) was collected during surgery (3 samples in each group), and stored at -80° C. The baseline characteristics of the subjects are shown in Table 2. The inclusion criteria for enrollment were as follows: 1) not participated in exercise training before 1 week of the surgery; 2) without any disease directly affecting skeletal muscle tissue morphology and/or function; 3) overall healthy. Total RNA was isolated from each individual sample with TRIzol reagent (Invitrogen, USA). The concentration and purity of total RNA were measured by Nanodrop2000 (Thermo Fisher, Waltham, MA, USA), and the integrity was detected using agarose gel electrophoresis.

**Table 2:** Baseline characteristics of young and older patients.

**Characteristics**	**Young adults (n = 3)**	**Older adults (n = 3)**	***P* value**
Age, y	33.00 ± 8.72	79.33 ± 0.58	< 0.001
Gender			0.37
Male	2	2	
Female	1	1	
Height, cm	168.70 ± 14.57	158.30±14.43	0.43
Weight, kg	68.00 ± 10.58	61.53 ± 18.02	0.62

### Library preparation for sequencing

Five μg RNA per sample was cleared for rRNA by beads Ribo-Zero Magnetic Kit (EpiCentre, Madison, WI, USA). The mRNA was randomly fragmented and single-strand cDNA was synthesized using random hexamer primer, the second strand cDNA synthesis was subsequently performed, and dTTP were replaced by dUTP. Then Illumina adaptor sequences were ligated to the end-repaired DNA fragments. The libraries were sequenced using the Hiseq2000 Truseq SBS Kit v3-HS (200 cycles).

### Quality control analysis of original sequencing data

Raw reads of fastq format were cleaned to remove empty reads, adapter sequences and fragments smaller than 25 bp, non-unique oligonucleotide (AGCT) reads at the 5' end, reads with over 10% N sequences, and low quality reads, in which the number of bases with a quality value Q ≤ 10 was > 50%. In addition, Q20%, Q30% and GC% of the clean data were calculated.

### Sequence alignment to reference genome and library quality assessment

Reads were mapped with Tophat (v2.0.9) to the human genome sequence (Ensemble GRCh38), and the Mapped Reads were analyzed with hisat2 (v 2.1.0, https://ccb.jhu.edu/software/hisat2/index.shtml). In addition, the sequence duplication, the non-uniform read distribution, the saturation for gene expression, the discreteness of insert sequence was evaluated with RSeQC (v2.6.4, http://rseqc.sourceforge.net/).

### Evaluation of mRNA expression

The Stringtie v1.3.3 package (http://ccb.jhu.edu/software/stringtie/) normally used to process read alignments and the reference annotation was applied to estimate gene expression level per million mapped reads (FPKM) score.

Pearson's Correlation Coefficient (r2) used as an indicator of correlations between two independent biological replicates was calculated by plot_cor_exp (v1.1.0). The closer r2 is to 1, the stronger the correlation between the two replicate samples.

### Analysis of DEGs

In the process of DEGs detection by edger (v3.24, http://www.bioconductor.org/packages/release/bioc/html/edgeR.html), |log2FC (fold change)| >1 and p value < 0.05 were used as screening criteria, and multi-test adjustment method (False Discovery Rate, FDR) was used to correct the p values. The clustering analysis of expression patterns was performed on DEGs using the distance calculation algorithm by plot_cluster_exp v1.1.0.

### Gene ontology (GO) analysis of DEGs

GO (Gene Ontology, http://www.geneontology.org/) database was used to classify genes according to the biological processes, cellular components and molecular functions. The p value was corrected by Bonferroni, Holm, Sidak and FDR approach. When the corrected p-value (FDR) < 0.05, it is considered that there is a significant enrichment of this GO function.

### KEGG pathway annotation of DEGs

To determine the most important biochemical metabolic pathways and signal transduction pathways involved in DEGs, the kegg_enrichment v2.1.0 was used with the Fisher's exact test. In order to control the false positive rate, the Benjamini–Hochberg (BH) procedure was used for multiple tests. KEGG pathway with corrected p value < 0.05 was defined as significantly enriched.

### Identification of DElncRNAs

The lncRNA expression level of FPKM score was calculated by Stringtie based on the gene annotation information of the lncRNA. In addition, the Pearson's r2 score between samples was calculated by plot_cor_exp. DElncRNAs with |log2FC (fold change)| > 1 and p value < 0.05 were screened, and the FDR controlled multi-test correction based on BH method was used to correct p values. The clustering analysis of expression patterns was performed on DElncRNAs using the distance calculation algorithm by plot_cluster_exp v1.1.0.

### DELncRNA-DEGs co-expression association analysis

Gene co-expression analysis could reveal the mechanism of transcriptional regulation. The interaction relationship was clarified via analyzing the PCC between DEGs and DElncRNAs in different samples. The interactions with PCC > 0.9 were filtered to construct a co-expression network using cytoscape software version 3.7.1.

### Cell culture and treatment

Human skeletal muscle myoblasts were purchased from LONZA Pharma and Biotech (Tokyo, Japan) and were cultured in DMEM containing 1% penicillin/streptomycin and 10% fetal bovine serum (Thermo Fisher Scientific, Waltham, MA, USA) at 37° C and 5% CO_2_. The medium was changed to high-glucose DMEM supplemented with 2% horse serum, 2% glutamine and 1% penicillin/streptomycin to induce myotubes. The medium was replaced every 48 h. On the fourth day after incubation with high-glucose DMEM, different concentrations of dexamethasone (Dex, 5 mM, 10 mM and 15 mM) were added and incubated for another 48 h. The expression of MyoD was determined by qRT-PCR to confirm the success of model establishment.

### Cell transfection

Skeletal muscle myoblasts were divided into four groups: control group (cells without treatment), model group (cells treated with Dex), si-NC (negative control) group (cells treated with Dex and transfected with negative control siRNA) and si-PRKG1-AS1 group (cells treated with Dex and transfected with siRNA-PRKG1-AS1). Si-NC and si-PRKG1-AS1 were synthesized by Guangzhou Ribobio biotechnology Co., Ltd (Guangdong, China). Skeletal muscle myoblasts were seeded into 24-well plate (1 × 10^5^/well). Si-NC (20 pM) or si-PRKG1-AS1 (20 pM) were transfected into cells at 70% confluence by 1 μL Lipofectamine 2000 (Thermo Fisher).

### Cell counting kit- 8 (CCK-8) assay

Skeletal muscle myoblasts were seeded on 96-well plate. 10 μ1 10% CCK-8 reagent was added to each well and incubated in the dark for 2 h. The absorbance at 450 nm was determined by a microplate reader (MK3, Thermo fisher).

### Flow cytometry analysis

Cell apoptosis was determined by flow cytometry (FACSCalibur, BD, San Jose, CA, USA) as described in previous study [50]. Cell apoptosis was determined by an Annexin V-FITC/Propidium Iodide (PI) apoptosis kit (BD, USA). Briefly, skeletal muscle myoblasts at 48 h post-transfection were centrifuged at 200 ×g for 5 min and re-suspended in 1× Binding buffer. Cell suspension (100 μL) was transferred into test tube, then FITC-Annexin V (5 μL) and PI (5 μL) were added and incubated at room temperature (25° C) for 15 min in dark. Cell apoptosis was tested on flow cytometry within 1h.

### qRT-PCR analysis for DElncRNA and DEGs

Tissue samples (50 mg) or skeletal muscle myoblasts of each group were extracted for total RNA by TRIzol reagent according to the manufacturer's instructions (TaKaRa, Dalian, China, Product code: 9109). Then reverse transcription reaction was performed for cDNA synthesis with PrimeScript™RT Master Mix (Perfect Real Time) (TaKaRa, Product code: RR036A). qRT-PCR was conducted to amplify genes using the following conditions: 50.0° C for 3min, 95.0° C for 3min, followed by 40 cycles of denaturation at 95.0° C for 10s and annealing-extension at 60.0° C for 30s. After reaction, the melting curve was evaluated by heating from 60° C to 95° C with 0.5° C for 10s increments. The primer sequences were shown in Table 3.

**Table 3:** The primer sequences for mRNAs and long non-coding RNAs (lncRNAs).

**Primers**	**Sequence (5’-3)**
SERPINE1-hF	AGTGGACTTTTCAGAGGTGGA
SERPINE1-hR	GCCGTTGAAGTAGAGGGCATT
OPRD1-hF	CGTCCGGTACACTAAGATGAAGA
OPRD1-hR	GCCACGTCTCCATCAGGTA
ITK-hF	GAAGATCGTCATGGGAAGAAGC
ITK-hR	CGGGTATTTATAGTGGCATGGG
TXNRD1-hF	ATATGGCAAGAAGGTGATGGTCC
TXNRD1-hR	GGGCTTGTCCTAACAAAGCTG
TDGF1-hF	CCCTCCTTCTACGGACGGAA
TDGF1-hR	CAGGGAACACTTCTTGGGCAG
CASC19-hF	CCTGGGTTAGAACCCTGCTG
CASC19-hR	TGGACAGCACCTTGAATGCT
AC103740.1-hF	GTTATGTGGCTTGCTGGTA
AC103740.1-hR	CTGGTCCTGAGTCACTTTGT
AC004797.1-hF	CTTGGCTTCGTTAGTGC
AC004797.1-hR	CTACTTCCTCCTCCTGTC
PRKG1-AS1-hF	CCTCCCTTGCTTAGTCGCTC
PRKG1-AS1-hR	TCTGCTATAACGCTCGCTGG
GPRC5D-AS1-hF	GCTGTGTGAGAACTCCGTGT
GPRC5D-AS1-hR	ACTATCAAAGGCAGGTCGGTG
GAPDH-hF	TGACAACTTTGGTATCGTGGAAGG
GAPDH-hR	AGGCAGGGATGATGTTCTGGAGAG
MIR1-1HG-AS1-hF	CCGTAAGACAACTCAGCATTAG
MIR1-1HG-AS1-hR	GGTTCTTGGACTGGGACGT
LINP1-hF	ATAATGTCCTCTACGTGCCG
LINP1-hR	CCCTCCTCCTTTCTTTGTG
MyoD-hF	CGCCATCCGCTATATCGAGG
MyoD-hR	CTGTAGTCCATCATGCCGTCG
MyoG-hF	GGGGAAAACTACCTGCCTGTC
MyoG-hR	AGGCGCTCGATGTACTGGAT
Mef2c-hF	GAACGTAACAGACAGGTGACAT
Mef2c-hR	CGGCTCGTTGTACTCCGTG
Myf5-hF	AACCCTCAAGAGGTGTACCAC
Myf5-hR	AGGACTGTTACATTCGGGCAT

### Western blot

Skeletal muscle myoblasts at 48 h post-transfection were added with RIPA lysis (Beyotime, Shanghai, China) to isolate total protein. Protein concentration was determined by BCA method (Thermo Fisher, USA), followed by separated on SDS-PAGE. Then, protein was transferred on to PVDF membrane (Millipore, USA), blocked with 5% skim milk and incubated with primary antibodies of anti-MyoD (Cal. No. 18943-1- AP, Proteintech, Rosemont, IL, USA; 1:1000), anti-MyoG (Cal. No. ab77232, Abcam, Cambridge, MA, USA; 1:1000), anti-Mef2c (Cal. No. 10056-1-AP, Proteintech, Rosemont, IL, USA; 1:1000), anti-Myf5 (Cal. No. ab125078, Abcam, Cambridge, MA, USA; 1:1000) and anti-GAPDH (Cal. No. 10494-1-AP, Proteintech, Rosemont, IL, USA; 1:1000) overnight at 4° C. On the second day, horseradish Peroxidase conjugated goat anti-rabbit IgG (H+L) (Cal. No. 111-035-003, Jackson ImmunoResearch, West Grove, PA) was added and incubated at 37° C for 2h. Chemiluminescence was developed by ECL system (Millipore, USA).

### Statistical analysis

All experiments were repeated for three times. Experimental data were expressed as mean ± standard deviation (SD) and were processed in Graphpad prism 5(Graphpad Software, San Diego, CA, USA). *P* < 0.05 represented statistical significance.

## Supplementary Material

Supplementary Table 1

Supplementary Table 2

Supplementary Table 3

Supplementary Table 4
